# Over-expression of Nectin-4 promotes progression of esophageal cancer and correlates with poor prognosis of the patients

**DOI:** 10.1186/s12935-019-0824-z

**Published:** 2019-04-23

**Authors:** Haifeng Deng, Hongbing Shi, Lujun Chen, You Zhou, Jingting Jiang

**Affiliations:** 1 Department of Tumor Biological Treatment, The Third Affiliated Hospital of Soochow University, Changzhou, 213003 Jiangsu China; 2 Research Center for Cancer Immunotherapy of Jiangsu Province, The Third Affiliated Hospital of Soochow University, Changzhou, 213003 Jiangsu China; 3 Institute of Cell Therapy, The Third Affiliated Hospital of Soochow University, Changzhou, 213003 Jiangsu China

**Keywords:** Nectin-4, Esophageal cancer, Cancer progression, Prognosis

## Abstract

**Background:**

Nectin-4, also known as PVRL4 (poliovirus-receptor-like 4), is specifically expressed in the embryo and placenta. Recent studies have reported that the Nectin-4 is over-expressed in multiple human cancers, and such abnormal expression is associated with cancer progression and poor prognosis of the patients. In the present study, we aimed to characterize the expression pattern of Nectin-4 in human esophageal cancer (EC) tissues, and to investigate its clinical implications, prognostic value and regulatory effects on cellular functions of EC cells.

**Methods:**

In the present study, we first examined Nectin-4 expression in human EC tissues by using immunohistochemistry (IHC) assay and analyzed the clinical associations. Then the cellular studies in vitro and the nude mice tumor model in vivo were used to examine the regulatory role of Nectin-4 in the progression of EC.

**Results:**

Our results demonstrated that over-expression of Nectin-4 in human EC tissues was significantly associated with tumor size, depth of tumor invasion, and poor prognosis of the patients. The intervention of Nectin-4 expression in EC cell lines showed that the increased Nectin-4 expression could significantly promote the cell viability, migration, invasion and tumor formation.

**Conclusions:**

Our present data unveiled that Nectin-4 played an important role in tumor biology and could serve as a useful prognostic predictor of human EC.

## Background

Esophageal cancer (EC) ranks the seventh in terms of incidence and the sixth in terms of mortality globally [1]. In China, the EC is also commonly diagnosed and identified as one of the most leading causes of cancer-related death [2]. According to histo-pathological classification, EC can be predominantly divided to squamous cell carcinoma (ESCC) and adenocarcinoma, and the ESCC accounts for nearly 90% of all EC cases [3]. Currently, the widely-used endoscopy screening and many therapeutic strategies have been available for EC, which greatly improve the overall survival rate of the patients. However, the local recurrence or distant metastasis still remains a major challenge for the treatment against EC. Therefore, it is urgently necessary to identify novel prognostic biomarkers and therapeutic targets for this malignancy.

It has been widely accepted that the loss of cell–cell adhesion contributes essentially to tumor initiation and progression [4]. Lots of cell-adhesion molecules are involved in this physio-pathological process, and such cell-adhesion molecules can be mainly divided into four groups, namely integrins, cadherins, selectins and immunoglobulin superfamily (IgSF) [5]. The nectins and nectin-like (necl) molecules are two important classes of cell-adhesion molecules within the IgSF family [6]. Nectin-4, also known as PVRL4 (poliovirus-receptor-like 4), is specifically expressed in the embryo and placenta, recent studies have reported that it is also over-expressed in several human cancers, including lung, gastric, ovarian and breast cancers, and the expression level of Nectin-4 in cancer tissues is significantly associated with cancer progression and poor prognosis of the patients [7–13]. Moreover, the abnormal expression of Nectin-4 in cancer cells could also regulate the cellular functions and angiogenesis [9]. Moreover, in addition to its membranous form, the soluble Nectin-4 can also be detected in serum from cancer patients, and therefore the serum level of soluble Nectin-4 can serve as important prognostic risk factor for the patients [8, 12].

In our present study, we aimed to characterize the expression pattern of Nectin-4 in human EC tissues, and to investigate its clinical implications and prognostic value. Moreover, we also performed in vitro and in vivo studies to further examine the effect of intervention of Nectin-4 expression on the cellular functions of EC cells. Our results demonstrated that over-expression of Nectin-4 in human EC tissues was significantly associated with tumor size, depth of tumor invasion, and poor prognosis of the EC patients. The intervention of Nectin-4 expression in EC cell lines could regulate the cell viability as well as the abilities of migration, invasion and tumor formation. Taken together, our present data unveiled that Nectin-4 played a crucial role in tumor biology, and it could serve as a useful prognostic predictor of human EC.

## Materials and methods

### Patients and tissue samples

The human EC tissue array (catalog: Heso-Squ172Sur-01) was purchased from Shanghai Outdo Biotech Co., Ltd. (Shanghai, P. R. China). A total of 94 patients who underwent surgery between July 2006 and October 2008 were enrolled in this study. Among these patients, 78 cases of adjacent normal tissues were also included in the tissue array. All these cancer tissues and adjacent normal tissues were confirmed using hematoxylin and eosin (H&E) staining after surgical resection. Incomplete tissue samples and several missing tissue samples were excluded during the heat-induced antigen retrieval, and finally a total of 82 cases were included in the present statistical analysis. Table 1 shows the detailed clinical parameters of the participants. All patients gave informed consent for participation, and the protocol for the present study was approved by the ethics committee of the Third Affiliated Hospital of Soochow University.

**Table 1:** Correlation between Nectin-4 expression in esophageal cancer tissues and patients’ clinical parameters

Clinical parameters	Cases	Nectin-4 expression level			*P*
		*H*-*score *< 70	*H*-*score *≥ 70	*χ* ^2^	
Age (years)				0.004	0.951
< 60	23	6	17		
≥ 60	59	15	44		
Tumor size (cm)				6.385	*0.012*
< 4.5	43	16	27		
≥ 4.5	39	5	34		
Gender				0.437	0.509
Male	62	17	45		
Female	20	4	16		
Lymph node metastasis				0.067	0.796
No	42	10	32		
Yes	38	10	28		
TNM stage				0.530	0.467
I–II	40	8	32		
III–IV	37	10	27		
Tumor stage				5.822	*0.016*
T1 + T2	15	7	8		
T3 + T4	63	11	52		

Italic signifies *P *< 0.05

### Immunohistochemical staining and evaluation

The immunostaining of Nectin-4 in EC tissue array was performed according to our published protocol [14, 15]. Briefly, the antigen retrieval was carried out by heating the tissue sections at 100 °C for 30 min in EDTA solution (pH 9.0). Goat anti-human Nectin-4 antibody (AF2659, R&D Systems) was used at the concentration of 10 μg/mL. The Polink-2 plus^®^ polymer HRP detection system for goat primary antibody (Zhongshan Golden Bridge Biotechnology, Beijing, China) was used according to the manufacturer’s instructions. Diaminobenzene was used as the chromogen, and hematoxylin was employed as the nuclear counterstain. The evaluation of Nectin-4 staining was performed according to the *H*-*score* method as described in our published reports [14–17].

### Cell culture

Human EC cell lines Eca-109 and TE-1 were obtained from Chinese Academy of Sciences, Shanghai Institutes for Biological Sciences. The cells were maintained in RPMI-1640 or DMEM supplemented with 10% FBS in the presence of benzylpenicillin (100 U/mL), streptomycin (100 μg/mL) and 2 mM l-glutamine, and were cultured under standard culture conditions (5% CO_2_, 37 °C).

### Nectin-4 over-expression, RNAi lentivirus generation, infection and cell sorting

The full-length of Nectin-4 (NM_030916.2; GenBank) fragment was synthesized by Sangon Biotech Shanghai Co., Ltd. (Shanghai, China) and cloned to pLV-IRES-ZsGreen-T2A-puro vector (Promega Biotech Co., Ltd., Madison, WI, USA). The small hairpin RNA (shRNA) against the human Nectin-4 gene was obtained from Shanghai GeneChem Co. Ltd (Shanghai, China), and cloned into a lentiviral gene transfer vector pLV-U6-GFP. The shRNA target sequence against Nectin-4 was 5′-CACTCCAAATACGGGCTTCAT-3′. The EC cell lines Eca-109 and TE-1 were transfected with LV-Nectin-4-shRNA, LV-NC, LV-Nectin-4-OE, and LV-Vector-Ctrl, and then selected using puromycin (2 µg/mL; Sigma-Aldrich; Merck Millipore) for more than 2 weeks.

### Real-time RT-PCR

Total RNA was extracted from EC cells, and the RNA quality was determined according to the methods as described in our previous studies [18]. The PCR reactions were performed on an ABI 7600 system (Applied Biosystems, USA) according to the manufacturer’s instructions. Human GAPDH was selected as a housekeeping gene. Primers were synthesized as follows, GAPDH forward primer: 5′-TGACTTCAACAGCGACACCCA-3′, GAPDH reverse primer: 5′-CACCCTGTTGCTGTAGCCAAA-3′; Nectin-4 forward primer: 5′-CTGAGCAGGTTCCCAGGTTT-3′, Nectin-4 reverse primer: 5′-AGAGTTCTTGCCTCTCGCAC-3′. The relative expression of Nectin-4 was calculated by the 2^−ΔΔCT^ method.

### Western blot analysis

The expression of Nectin-4 at the protein level in different cellular models was determined by Western blotting analysis according to the protocol described in our published reports [14, 15].

### Cell viability assay

The effects of Nectin-4 intervention on biological functions of EC cell lines were assessed according to our previously published protocols [14, 15]. Cell viability was assessed using Cell Counting Kit-8 (CCK-8, Beyotime, Shanghai, China) according to the manufacturer’s instructions. Briefly, 5 × 10^3^ Eca-109 or TE-1 cells from LV-Nectin-4-shRNA, LV-NC, LV-Nectin-4-OE and LV-Vector-Ctrl were seeded into 96-well plates and incubated for 24, 48 and 72 h. CCK-8 reagent was added to each well at 3 h before the endpoint of incubation, and the absorbance of each well was determined at a wavelength of 450 nm by a microplate reader. An increase or decrease in the absorbance of experimental wells relative to the initial values indicates cell growth or death, respectively. Each experiment was repeated for at least three times.

### Wound healing assay

Eca-109 or TE-1 cells from LV-Nectin-4-shRNA, LV-NC, LV-Nectin-4-OE and LV-Vector-Ctrl groups were cultured in 6-well plates. A small wound area was created using a 200-µL pipette tip when cells reached a 90% confluence. Cells were washed twice with PBS and then incubated in serum-free RPMI-1640 or DMEM medium at 37 °C for 48 h in a 5% CO_2_ incubator. Photographs were acquired at two different time points (0 and 24 h). Wound width was measured using a BX50 microscope (Olympus^®^) with a calibrated eyepiece grid. Data from three independent experiments were averaged and expressed as a percentage of the original width.

### Invasion assay

The invasion assay was used to evaluate the effect of intervention of Nectin-4 expression on the invasion ability of human esophageal cancer cells as previously described [12, 13]. Briefly, cells from the different groups were placed in the upper chamber of Matrigel-coated invasion chamber (Corning, NY, USA) and serum-starved for 24 h, and then the medium containing 10% FBS was placed in the lower chamber as a chemo-attractant. After 48 h of incubation, those cells that migrated into the lower chamber were collected and re-suspended, and non-migrating cells were removed from the top of the Matrigel with a cotton-tipped swab. Migrated cells were fixed and stained with 0.1% crystal violet and later photographed under a microscope (Olympus, Tokyo, Japan). Finally, the migrated cells were counted from five randomly selected fields.

### Cell cycle assay

The Eca-109 or TE-1 cells from LV-Nectin-4-shRNA, LV-NC, LV-Nectin-4-OE and LV-Vector-Ctrl groups were cultured in 6-well plates and cultured for 48 h. The cells were then washed with ice-cold PBS and fixed in a 70% (v/v) ice-cold ethanol solution at 4 °C overnight. Subsequently, these cells were analyzed by flow cytometry according to the instructions of cell cycle analysis kit (Sigma, MO, USA). The cell cycle information was analyzed using ModFit LT 4.0 software.

### Subcutaneous transplantation model study

The subcutaneous transplantation model study was performed according to our previously published protocol [19]. Briefly, eight groups of female Balb/c nude mice (4–6 weeks old, five mice for each group) were bred under a specific-pathogen-free (SPF) condition with constant humidity and temperature (25–28 °C). Animal protocols were approved by the Animal Care and Use Committee and carried out in compliance with the Guidelines on Animal Welfare of the China National Committee for Animal Experiments. Eca-109 cells (1 × 10^7^) or TE-1 cells (1 × 10^7^) from LV-Nectin-4-shRNA, LV-NC, LV-Nectin-4-OE and LV-Vector-Ctrl groups were suspended in 0.15 mL PBS, and cell suspension was subcutaneously injected into the right back region of nude mice. The tumor size was measured every 2 days with caliper, and the volume was calculated using the formula as follows: Length × Width^2^ × π/6. Growth curves were constructed, and the data were presented as mean ± SEM. Finally, the tumors were harvested from mice at 28 days post injection. The protocol for the tumor model by using nude mice in the present study was approved by the ethics committee of the Third Affiliated Hospital of Soochow University.

### Statistical analyses

Statistical analysis was completed using the paired Student’s *t*-test, the Wilcoxon signed rank test, the two way ANOVA analysis, the Chi square test or the Log-rank survival analysis where appropriate for final analysis of the data. All the statistical analyses were performed using the GraphPad Prism 5.0 software package (GraphPad Software, Inc., San Diego, USA). A *P*-value of < 0.05 was considered as statistically significant.

## Results

### Survey of Nectin-4 expression at mRNA level in human EC tissues based on TCGA data

**Fig. 1:**
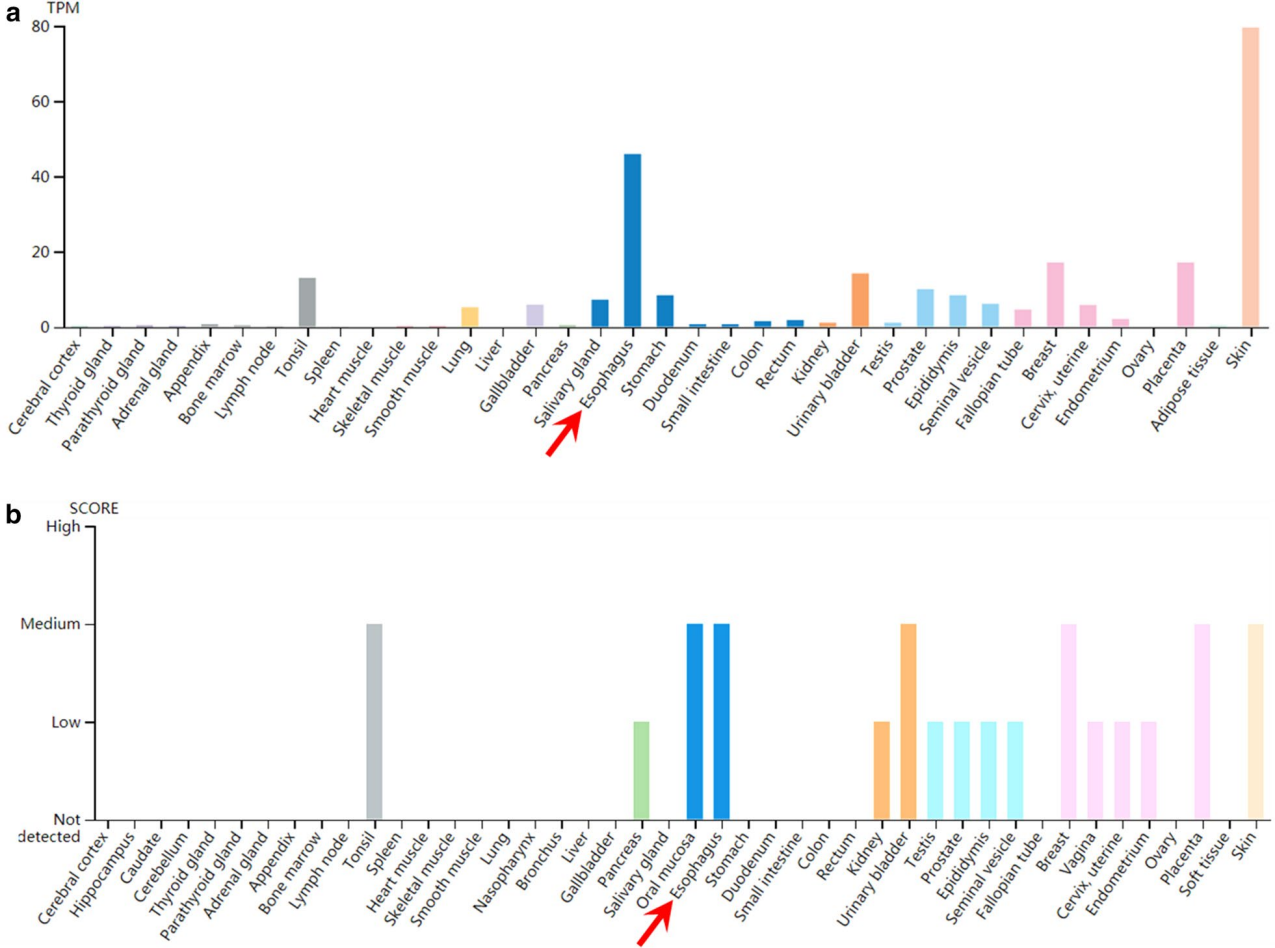
Nectin-4 expression profiles in normal human tissues. **a**, **b** The RNA (**a**) and protein (**b**) expression profiles of Nectin-4 in normal human tissues. Data credit: Human Protein Atlas. Data summary images were obtained from: https://www.proteinatlas.org/ENSG00000143217-NECTIN4/tissue

**Fig. 2:**
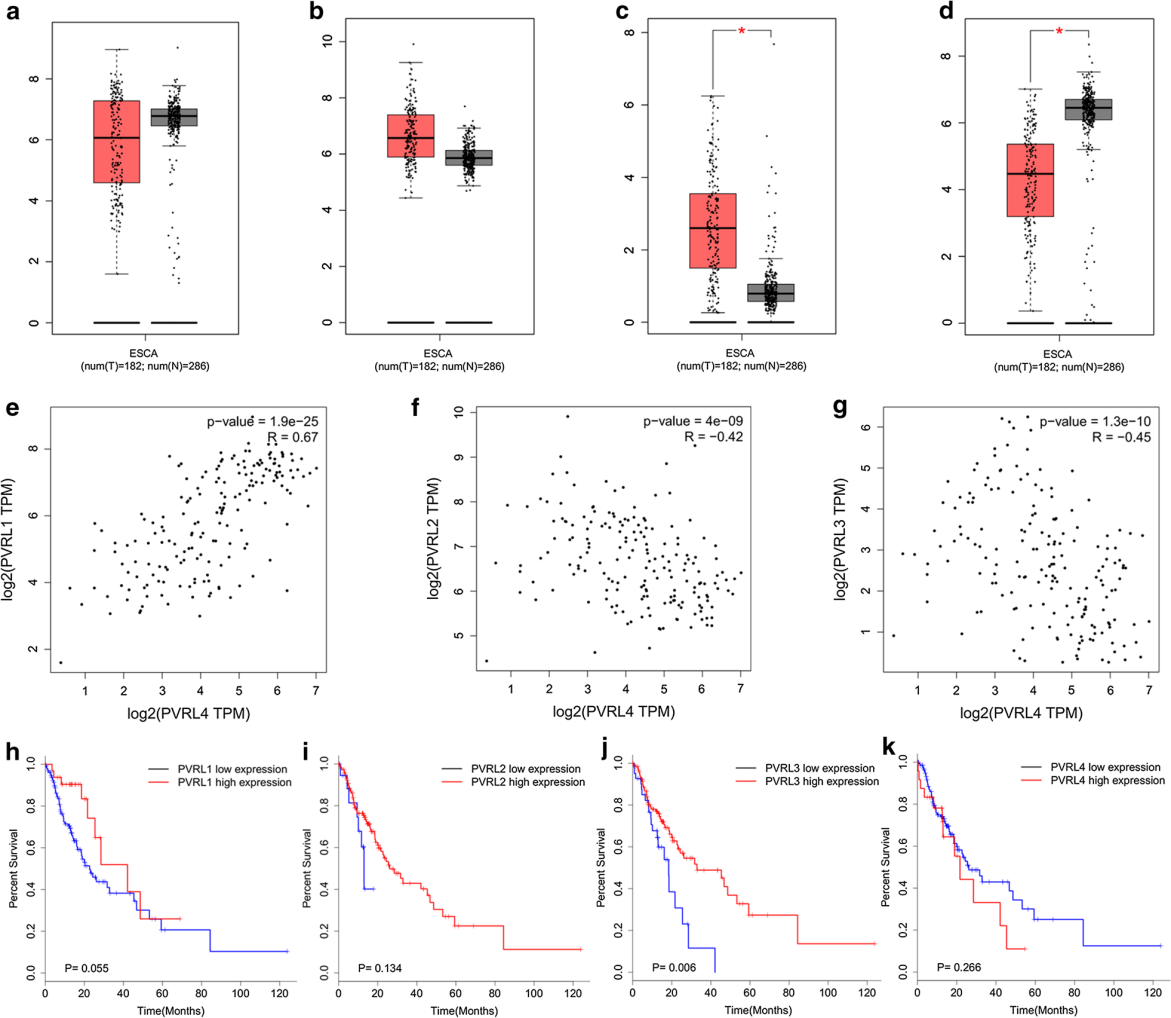
Survey of Nectin-4 expression at mRNA level in human EC tissues based on TCGA data. **a**, **b** Based on the TCGA data from http://gepia.cancer-pku.cn/, we could not find any significant difference of Nectin-1 and Nectin-2 at mRNA level between cancer tissues and adjacent normal tissues. **c**, **d** We found that the mRNA expression level of Nectin-3 was significantly higher in cancer tissues compared with that in adjacent normal tissues (*P *< 0.05), and the mRNA expression level of Nectin-4 was significantly higher in adjacent normal tissues compared with cancer tissues (*P *< 0.05). **e**–**g** We found that the expression of Nectin-4 at the mRNA level in EC tissues was positively and significantly correlated with that of Nectin-1 (*P *< 0.0001), while it was negatively and significantly correlated with that of Nectin-2 (*P *< 0.0001) and Nectin-3 (*P *< 0.0001). **h**, **i** We didn’t find any significant correlations between patients’ postoperative survival and Nectin-1 or Nectin-2. **j** The overall survival rate of the patients with higher expression of Nectin-3 at the mRNA level was significantly greater compared with those showing lower Nectin-3 expression (*P *= 0.006). **k** The patients with lower expression of Nectin-4 at the mRNA level exhibited better overall survival rate compared with those showing higher Nectin-4 expression, although the difference was not significant (*P *= 0.266)

According to the data from https://www.proteinatlas.org, we characterized Nectin-4 expression in different normal human tissues, and the results revealed that Nectin-4 expression could found in normal esophagus tissues at both mRNA (Fig. 1a) and protein (Fig. 1b) levels. Moreover, based on the TCGA data from http://gepia.cancer-pku.cn/, we studied the mRNA expression levels of Nectin family members including Nectin-1, -2, -3 and -4 in human EC tissues, Fig. 2c shows that the mRNA expression level of Nectin-3 was significantly higher in cancer tissues compared with that in adjacent normal tissues (*P *< 0.05). Figure 2d illustrates that the mRNA expression level of Nectin-4 was significantly higher in adjacent normal tissues compared with cancer tissues (*P *< 0.05), while there were no any significant differences in terms of the expressions of Nectin-1 and Nectin-2 between cancer tissues and adjacent normal tissues (Fig. 1a, b). Then we further examined the correlations between the expressions of Nectin-4 in EC tissues and other members namely Nectin-1, -2 and -3. Our results demonstrated that the expression of Nectin-4 at the mRNA level in EC tissues was positively and significantly correlated with that of Nectin-1 (*P *< 0.0001, Fig. 2e), while it was negatively and significantly correlated with that of Nectin-2 (*P *< 0.0001, Fig. 2f) and Nectin-3 (*P *< 0.0001, Fig. 2g). Furthermore, we also examined the prognostic value of Nectin family members in human EC tissues based on TCGA data. We didn’t find any significant correlations between patients’ postoperative survival and Nectin-1 or Nectin-2 (Fig. 2h, i). Figure 2j reveals that the overall survival rate of the patients with higher expression of Nectin-3 at the mRNA level was significantly greater compared with those showing lower Nectin-3 expression (*P *= 0.006). Moreover, the patients with lower expression of Nectin-4 at the mRNA level exhibited better overall survival rate compared with those showing higher Nectin-4 expression, although the difference was not significant (Fig. 2k, *P *= 0.266).

### Nectin-4 expression in human EC tissues and its clinical implications

**Fig. 3:**
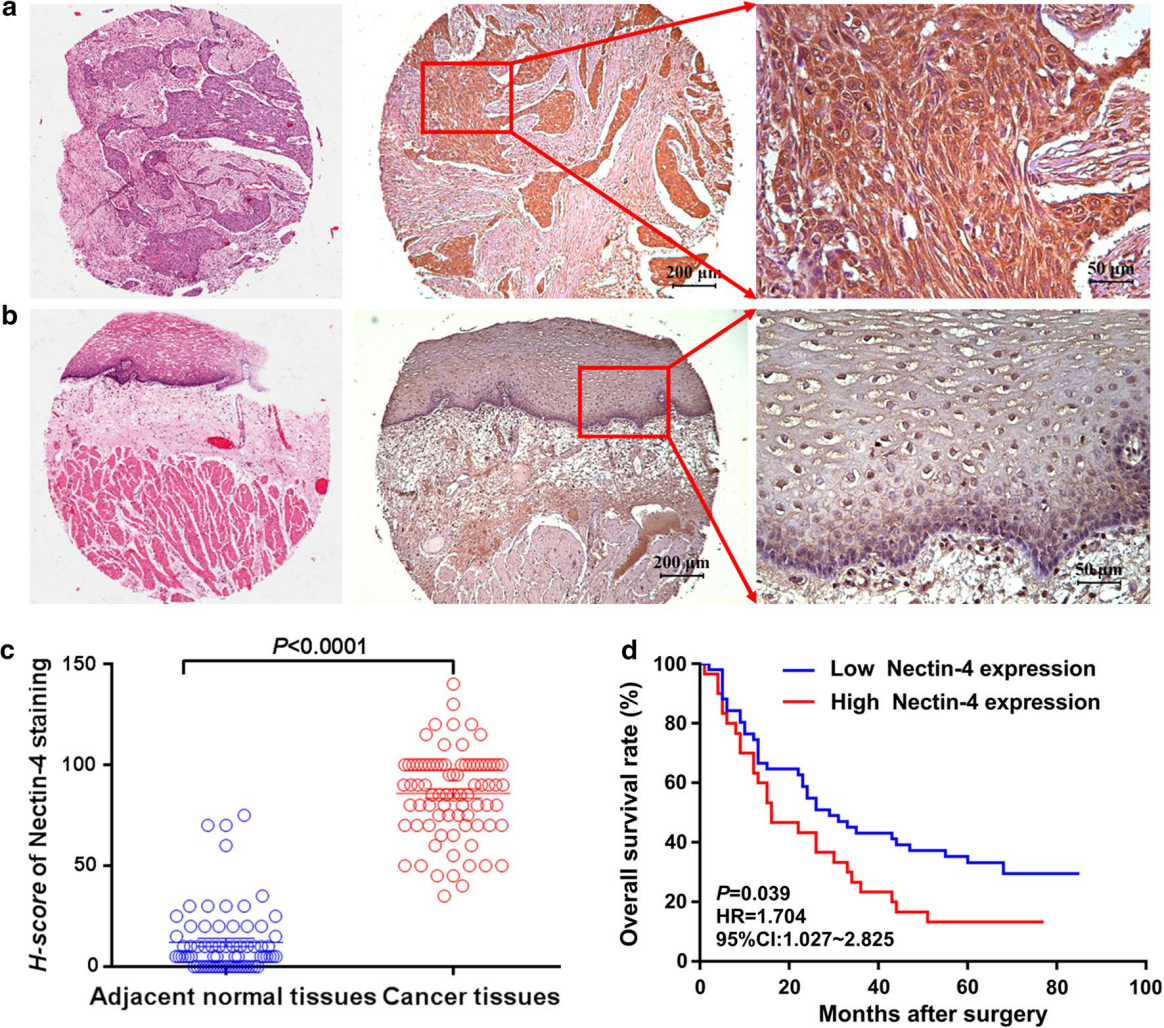
Nectin-4 expression in EC tissues. **a**, **b** Positive staining of Nectin-4 could be found in the cytoplasm and on the membrane of cancer cells, while weak or negative staining of Nectin-4 was found in normal esophageal tissues. **c** The staining intensity of Nectin-4 in EC tissues was significantly higher than that in adjacent normal tissues (*P *< 0.0001). **d** The survival analysis showed that the overall survival rate of the patients with higher Nectin-4 expression was significantly poorer compared with those showing lower Nectin-4 expression (HR = 1.704, 95% CI 1.027–2.825, *P *= 0.039)

In order to further investigate the clinical significance of Nectin-4 expression in human EC, we carried out the immunohistochemistry to study the expression of Nectin-4 in EC tissues. Figure 3a shows that positive staining of Nectin-4 could be found in the cytoplasm and on the membrane of the cancer cells, while weak or negative staining of Nectin-4 could be found in normal esophageal tissues (Fig. 3b). Figure 3c reveals that the staining intensity of Nectin-4 in EC tissues was significantly higher than that in adjacent normal tissues (*P *< 0.0001). Moreover, the survival analysis showed that the overall survival rate of the patients with higher Nectin-4 expression was significantly poorer compared with those showing lower Nectin-4 expression (HR = 1.704, 95% CI 1.027–2.825, *P *= 0.039) (Fig. 3d).

Besides, we also analyzed the associations between the expression of Nectin-4 in EC tissues and patient’s clinical parameters. Table 1 shows that the staining intensity of Nectin-4 was positively and significantly associated with tumor size (*P *= 0.012) and tumor stage (*P *= 0.016). According to the COX model analysis, the Nectin-4 expression level could serve as an independent prognostic predictor for EC patients (Table 2, HR = 1.795, 95% CI 1.042–3.092, *P *= 0.035), suggesting that abnormal expression of Nectin-4 was involved in the progression of EC.

**Table 2:** Cox model analysis for the correlation between Nectin-4 expression level and patients’ clinical parameters

Clinical parameters	Uni-variate		Multi-variate	
	HR (95% *CI*)	*P*	HR (95% *CI*)	*P*
Gender (M/F)	2.618 (1.285–5.337)	0.008	1.827 (0.765–4.364)	0.175
Age (years) (≥ 60/< 60)	1.093 (0.630–1.894)	0.752	1.543 (0.828–2.878)	0.172
Tumor size (≥ 4.5 cm/< 4.5 cm)	1.674 (1.014–2.763)	0.044	1.363 (0.792–2.346)	0.264
Pathological stage (III–IV/I–II)	0.805 (0.436–1.485)	0.487	0.723 (0.377–1.385)	0.328
Tumor stage (T3–T4/T1–T2)	2.950 (0.923–7.127)	0.008	1.613 (0.898–5.021)	0.087
Lymph node metastasis (yes/no)	2.518 (1.290–3.609)	0.003	1.689 (0.928–3.074)	0.086
Nectin-4 expression (high/low)	1.704 (1.027–2.825)	0.039	1.795 (1.042–3.092)	0.035

### Intervention of Nectin-4 expression in human EC cells

**Fig. 4:**
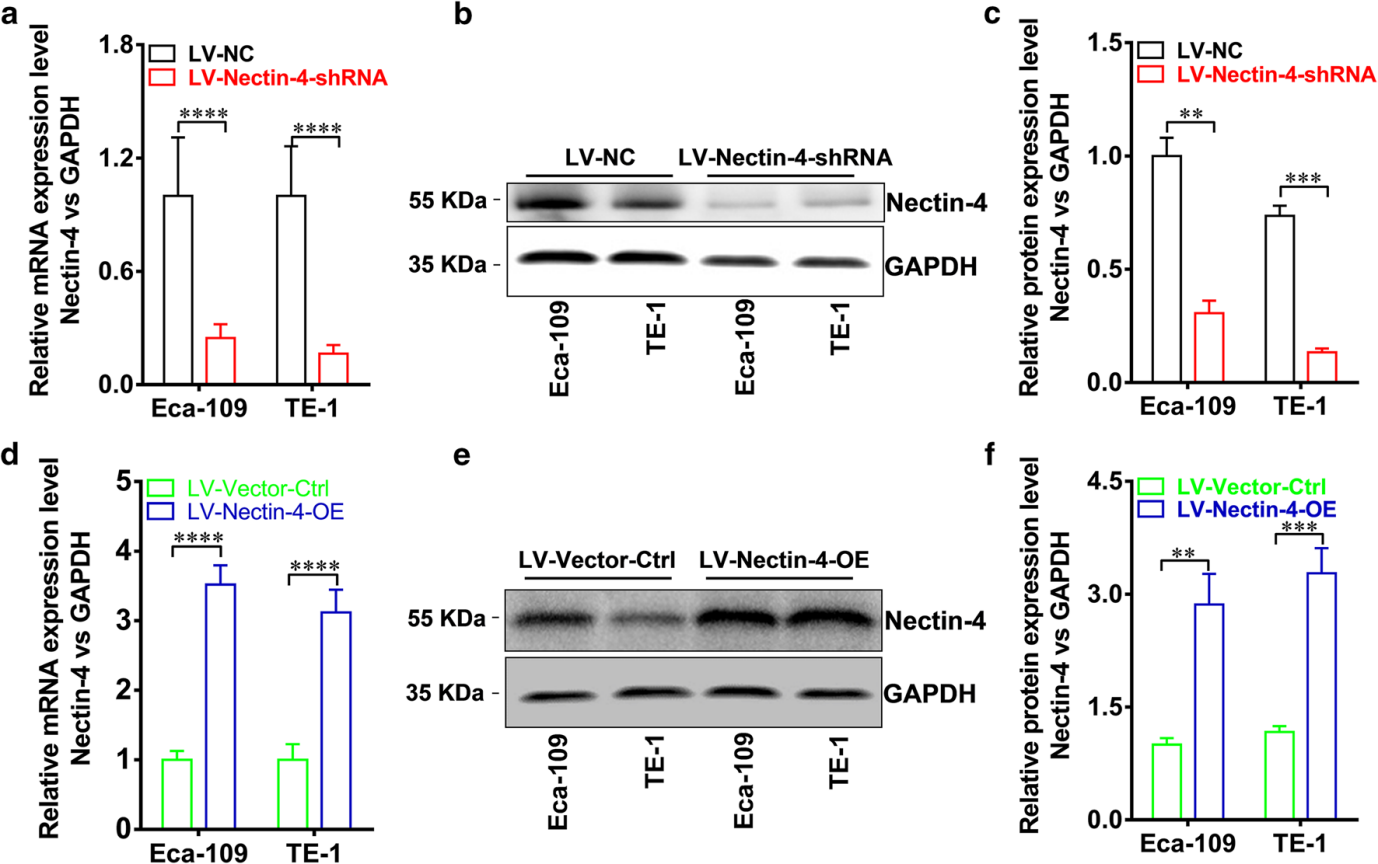
Knockdown expression and over-expression of Nectin-4 in human EC cell lines Eca-109 and TE-1. **a** The knockdown expression of Nectin-4 at mRNA level by using RNAi approach in human EC lines was confirmed by real-time RT-PCR, and the Nectin-4 mRNA expression level in LV-Nectin-4-shRNA group cells was significantly lower than that in LV-NC group cells (both in Eca-109 and in TE-1, *P *< 0.0001). **b** The decreased Nectin-4 protein expression after knockdown in human EC cell lines Eca-109 and TE-1, were confirmed by using western blot method. **c** The Nectin-4 protein expression level in LV-Nectin-4-shRNA group cells was significantly lower than that in LV-NC group cells (in Eca-109, *P *< 0.01, and in TE-1, *P *< 0.001). **d** The over-expression of Nectin-4 at mRNA level in human EC lines was confirmed by real-time RT-PCR, which showed that the increased Nectin-4 mRNA expression level in LV-Nectin-4-OE group cells compared with LV-Vector-Ctrl group cells (both in Eca-109 and in TE-1, *P *< 0.0001). **e** The increased Nectin-4 protein expression in human EC cell lines Eca-109 and TE-1 were confirmed by using western blot method. **f** The Nectin-4 protein expression level in LV-Nectin-4-OE group cells was significantly higher than that in LV-Vector-Ctrl group cells (in Eca-109, *P *< 0.01, and in TE-1, *P *< 0.001)

In order to further investigate whether the intervention of Nectin-4 expression in human EC cells had effects on cellular functions, we also carried out the cellular study on the knock-down expression or over-expression of Nectin-4 in EC cells. Figure 4 shows that Nectin-4-knockdown expression or Nectin-4-over-expressing cell lines were successfully established using lentiviral transfection, and both real-time RT-PCR and Western blotting analyses were used to confirm the intervention of Nectin-4 expression in human EC cells at the mRNA and protein levels, respectively.

### Effect of intervention of Nectin-4 expression on cellular functions of human EC cell lines

**Fig. 5:**
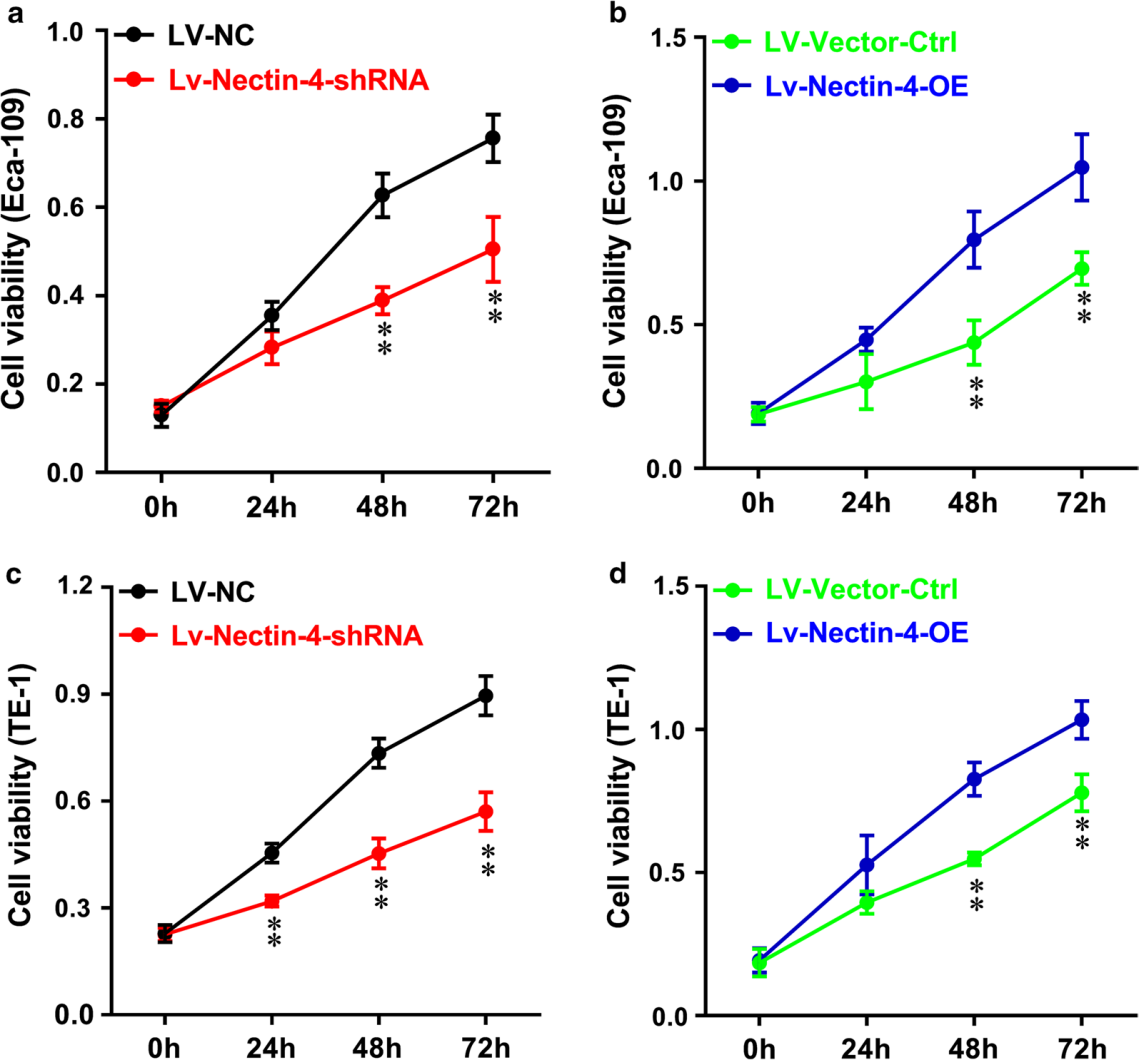
Effect of intervention of Nectin-4 expression on cell proliferation of human EC cell lines. We examined the intervention of Nectin-4 expression on the cell proliferation in vitro using CCK-8 assay in human EC cell lines. **a** In Eca-109 cells, at 48 and 72 h after seeding, the cell proliferation rate of LV-Nectin-4-shRNA group was significantly lower compared with the LV-NC group (both *P *< 0.01). **b** In Eca-109 cells, at 48 and 72 h after seeding, the cell proliferation rate of LV-Nectin-4-OE group was significantly higher compared with the LV-Vector-Ctrl group (both *P *< 0.01). **c** In TE-1 cells, at 24, 48 and 72 h after seeding, the cell proliferation rate of LV-Nectin-4-shRNA group was significantly lower compared with the LV-NC group (all *P *< 0.01). **d** In TE-1 cells, at 48 and 72 h after seeding, the cell proliferation rate of LV-Nectin-4-OE group was significantly higher compared with the LV-Vector-Ctrl group (both *P *< 0.01)

**Fig. 6:**
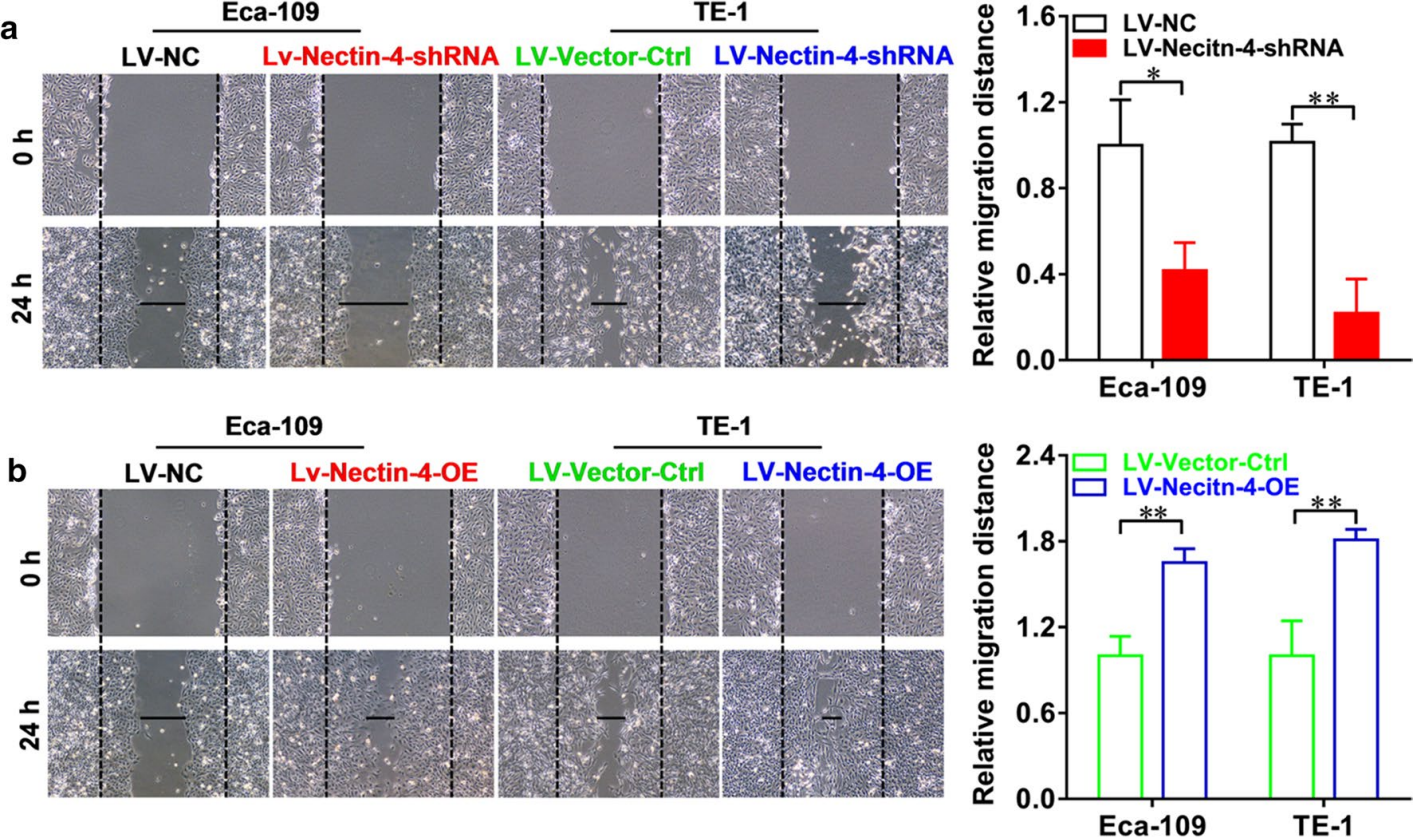
The intervention of Nectin-4 expression affects the migration ability of human EC cell lines. The wound healing assay was performed to evaluate the effect of the intervention of Nectin-4 expression on the migration ability of human EC cell lines. **a** Knockdown of Nectin-4 expression significantly decreased the cell migration ability of Eca-109 as well TE-1 cells, the cell-free area of LV-Nectin-4-shRNA group was significantly wider than that of LV-NC group at 24 h (in Eca-109, *P *< 0.05, and in TE-1, *P *< 0.01). **b** Over-expression of Nectin-4 expression significantly increased the cell migration ability of Eca-109 as well TE-1 cells, the cell-free area of LV-Nectin-4-OE group was significantly narrower than that of LV-Vector-Ctrl group at 24 h (both *P *< 0.01)

**Fig. 7:**
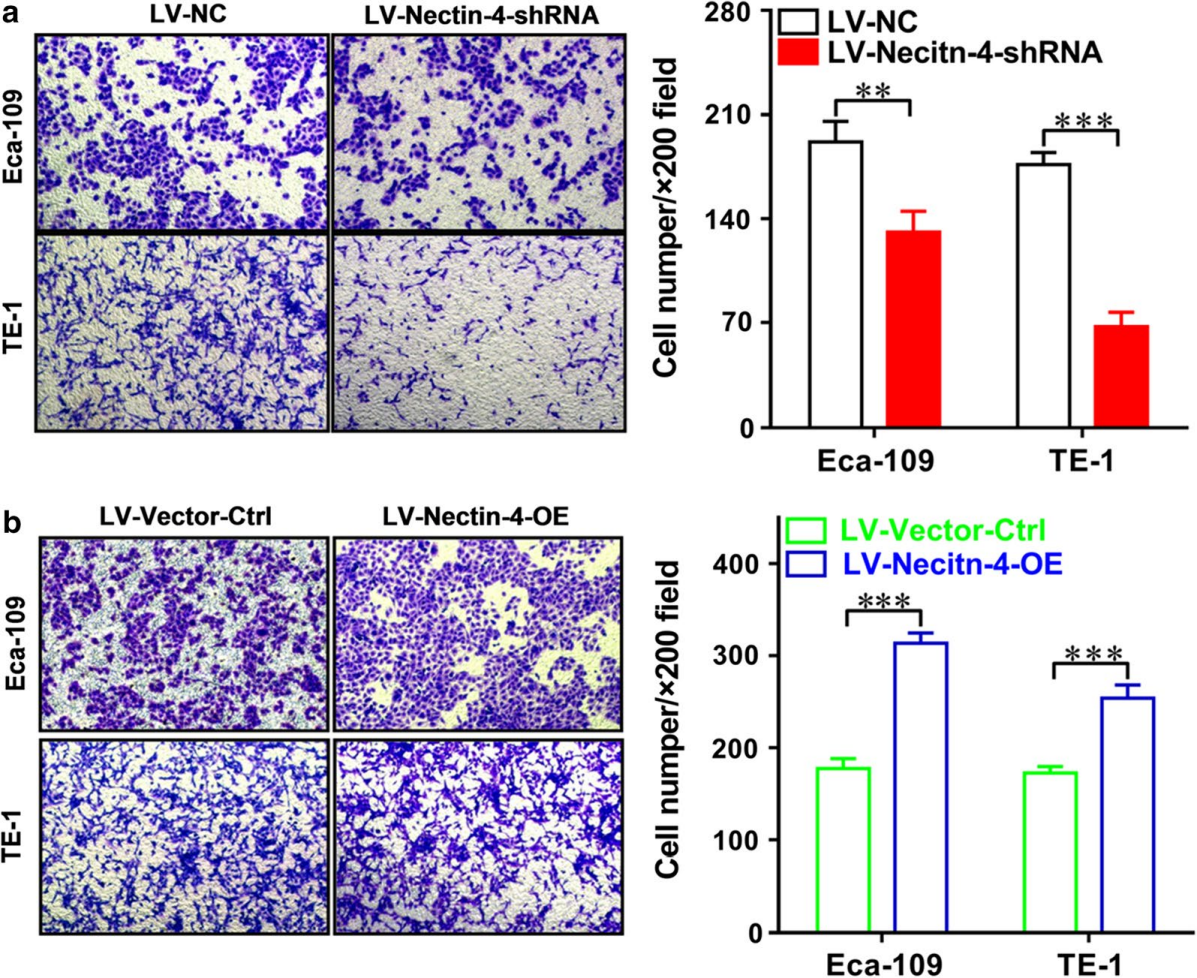
The intervention of Nectin-4 expression affects the invasion ability of human EC cell lines. The transwell invasion assay was performed to evaluate the effect of the intervention of Nectin-4 expression on the invasion ability of human EC cell lines. **a** Knockdown of Nectin-4 expression significantly decreased the number of invaded cells stained with Cristal Violet in LV-Nectin-4-shRNA group cells compared with LV-NC group cells (in Eca-109, *P *< 0.01, and in TE-1, *P *< 0.001). **b** Nectin-4 over-expression significantly increased the number of invaded cells stained with Cristal Violet in LV-Nectin-4-OE group cells compared with LV-Vector-Ctrl group cells (both *P *< 0.001)

We then examined the effects of the intervention of Nectin-4 expression on the cell proliferation in vitro by using CCK-8 assay in human EC cell lines. As shown in Fig. 5a, in Eca-109 cells, at 48 as well as 72 h after seeding, the proliferation rate of LV-Nectin-4-shRNA group cells was significantly lower than that of LV-NC group cells (both *P *< 0.01). Figure 5b shows that, in Eca-109 cells, at 48 as well as 72 h after seeding, the proliferation rate of LV-Nectin-4-OE group cells was significantly higher than that of LV-Vector-Ctrl group cells (both *P *< 0.01). As shown in Fig. 5c, in TE-1 cells, at 24, 48 as well as 72 h after seeding, the proliferation rate of LV-Nectin-4-shRNA group cells was significantly lower than that of LV-NC group cells (*P *< 0.01 respectively). Figure 5d shows that in TE-1 cells, at 48 as well as 72 h after seeding, the proliferation rate of LV-Nectin-4-OE group cells was significantly higher than that of LV-Vector-Ctrl group cells (both *P *< 0.01). Moreover, the wound-healing assay was performed to evaluate the effect of the intervention of Nectin-4 expression on the migration ability of human EC cell lines. Knockdown of Nectin-4 expression significantly decreased the cell migration ability of Eca-109 and TE-1 cells, showing that the cell-free area of the LV-Nectin-4-shRNA group was significantly wider than that of the LV-NC group at 24 h (Fig. 6a, in Eca-109, *P *< 0.05, and in TE-1, *P *< 0.01). Over-expression of Nectin-4 expression significantly increased the cell migration ability of Eca-109 and TE-1 cells, showing that the cell-free area of LV-Nectin-4-OE group was significantly narrower than that of the LV-Vector-Ctrl group at 24 h (Fig. 6b, both *P *< 0.01). Furthermore, the transwell invasion assay was performed to evaluate the effect of the intervention of Nectin-4 expression on the invasion ability of human EC cell lines. Knockdown of Nectin-4 expression significantly decreased the number of invaded cells stained with Cristal Violet in the LV-Nectin-4-shRNA group cells compared with the LV-NC group cells (Fig. 7a, in Eca-109, *P *< 0.01, and in TE-1, *P *< 0.001). Nectin-4 over-expression significantly increased the number of invaded cells stained with Cristal Violet in the LV-Nectin-4-OE group cells compared with the LV-Vector-Ctrl group cells (Fig. 7b, both *P *< 0.001).

### Effect of Nectin-4 intervention on tumor growth in the subcutaneous transplantation mouse model

**Fig. 8:**
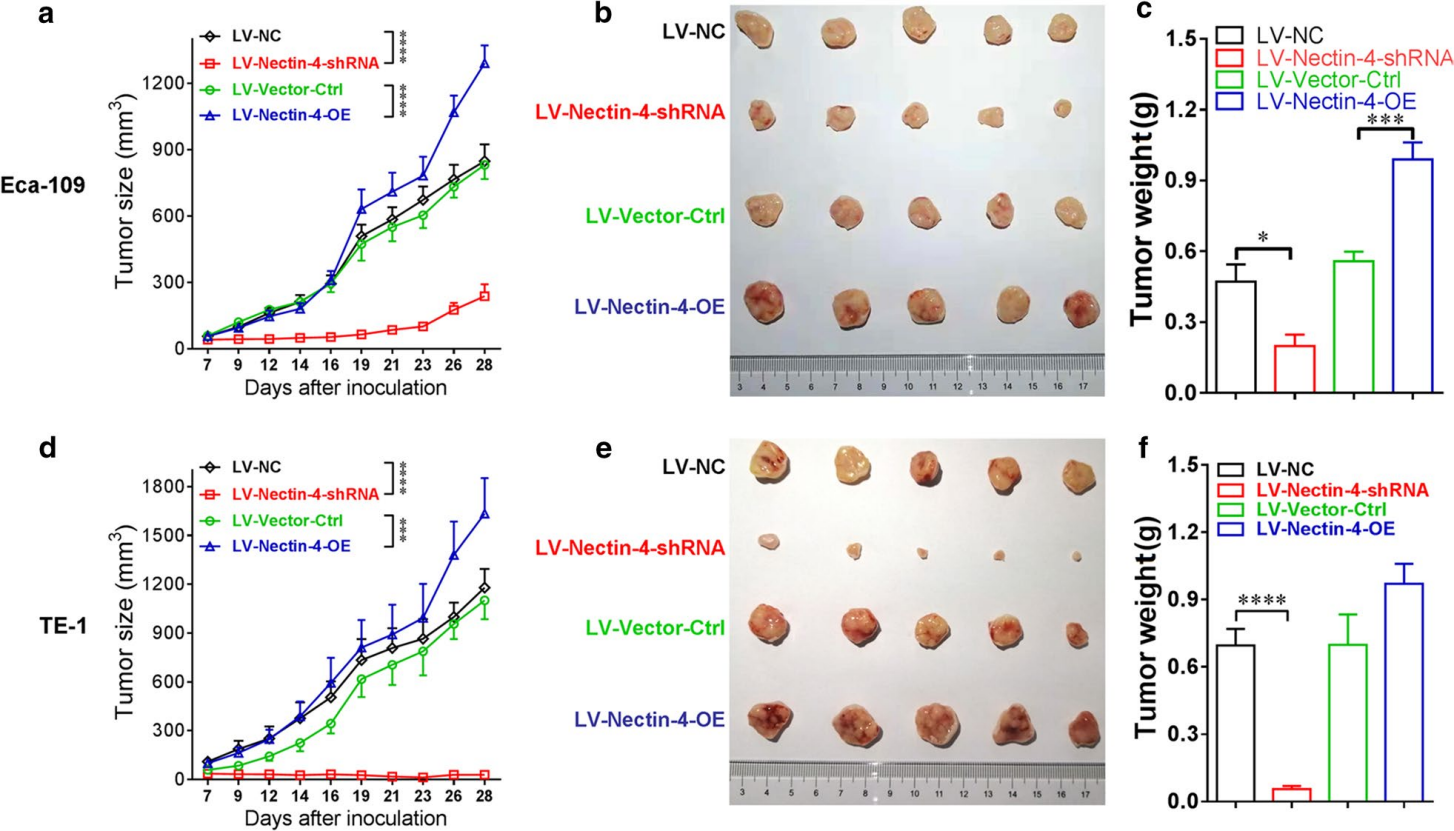
Intervention of Nectin-4 expression effects the tumor growth in the subcutaneous transplantation mouse model. The subcutaneous transplantation mouse model was used to evaluate the effect of the intervention of Nectin-4 expression on the tumor growth of human EC cell lines. The Eca-109 or TE-1 cells from LV-NC, LV-Nectin-4-shRNA, LV-Vector-Ctrl and LV-Nectin-4-OE groups were injected subcutaneously into nude mice (n = 5, respectively). **a** In tumor models established by using Eca-109 cells, the tumor growth curves showed that knockdown of Nectin-4 expression significantly inhibits tumor growth (*P *< 0.0001), and the Nectin-4 over-expression significantly promotes tumor growth (*P *< 0.0001). **b** The tumor tissues resected from the tumor models established by Eca-109 cells from each groups were listed. **c** After 28 days, in tumor models established by using Eca-109 cells, the tumor weight of LV-Nectin-4-shRNA group was lighter than that of LV-NC group (*P *< 0.05), the tumor weight of LV-Nectin-4-OE group trended heavier than that of LV-Vector-Ctrl group (*P *< 0.001). **d** In tumor models established by using TE-1 cells, the tumor growth curves showed that knockdown of Nectin-4 expression significantly inhibits tumor growth (*P *< 0.0001), and the Nectin-4 over-expression significantly promotes tumor growth (*P *< 0.001). **e** The tumor tissues resected from the tumor models established by TE-1 cells from each groups were listed. **f** After 28 days, in tumor models established by using TE-1 cells, the tumor weight of LV-Nectin-4-shRNA group was lighter than that of LV-NC group (*P *< 0.0001), the tumor weight of LV-Nectin-4-OE group trended heavier than that of LV-Vector-Ctrl group (*P *= 0.1314)

In order to study the effect of abnormal Nectin-4 expression on the regulation of tumor growth in vivo, we then established the subcutaneous transplantation mouse model. Eca-109 or TE-1 cells of LV-Nectin-4-shRNA, LV-NC, LV-Nectin-4-OE and LV-Vector-Ctrl groups were subcutaneously injected into nude mice. In the tumor model established using Eca-109 cells, the tumor growth curves showed that knockdown expression of Nectin-4 significantly inhibited the tumor growth (Fig. 8a, *P *< 0.0001), while the tumor growth of Nectin-4 over-expression group also trended to be faster than that of control group (Fig. 8a, *P *= 0.0831). After 28 days, the tumor weight of the LV-Nectin-4-shRNA group was lighter compared with the LV-NC group (Fig. 8c, *P *< 0.05), and the tumor weight of the LV-Nectin-4-OE group trended to be heavier compared with the LV-Vector-Ctrl group (Fig. 8c, *P *< 0.001). In the tumor model established using TE-1 cells, the tumor growth curves showed that knockdown expression of Nectin-4 significantly inhibited the tumor growth (Fig. 8d, *P *< 0.001), while the tumor growth of Nectin-4 over-expression group also trended to be faster than that of control group (Fig. 8d, *P *= 0.1816). After 28 days, the tumor weight of the LV-Nectin-4-shRNA group was lighter compared with the LV-NC group (Fig. 8f, *P *< 0.0001), and the tumor weight of the LV-Nectin-4-OE group trended to be heavier compared with the LV-Vector-Ctrl group (Fig. 8f, *P *= 0.1314).

## Discussion

Esophageal cancer is one of the most common malignant tumors of the digestive system, especially in China [20–22]. Although some improvements have been made in the diagnosis and treatment of EC, the 5-year survival rate of the patients with advanced EC still remains less than 15% due to lymph node invasion and distant metastasis [20, 21]. Therefore, it is of great importance to clarify the molecular mechanism underlying the oncogenesis and development of EC in order to optimize the therapeutic strategies and to improve the prognosis of EC patients. In our present study, we focused on the clinical implications of Nectin-4 expression in human EC tissues and further revealed its potential regulatory role in EC cells. Our results demonstrated that Nectin-4 expression was significantly higher in cancer tissues compared with the adjacent normal tissues, and such up-regulation in cancer tissues was significantly correlated with advanced tumor stage and poorer prognosis of the EC patients. Moreover, our cellular study and in vivo study also showed that the intervention of Nection-4 in human EC cells could regulate the tumor growth and cellular functions, such as viability, migration ability, invasive ability and cell cycle, suggesting that abnormal expression of Nectin-4 was involved in the cancer progression in this malignancy.

Several clinical investigations have revealed that Nectin-4 can serve as a tumor biomarker, and its over-expression in cancer tissues is significantly associated with cancer progression and poorer prognosis of the patients [9, 11–13, 20, 23–25]. It has been demonstrated that higher Nectin-4 expression is found in human gastric cancer tissues compared with the normal gastric tissues, and the expression level of Nectin-4 is significantly associated with cancer cell differentiation, lymph node metastasis, advanced TNM stage and poorer prognosis of the patients [13]. The abnormal expressions of both membranous and soluble forms of Nectin-4 have been found in human breast cancer tissues and sera from the patients. Furthermore, the levels of both two forms of Nectin-4 can be used as important biomarkers and prognostic predictors for breast cancer patients [11, 23, 24]. In human pancreatic carcinoma, the over-expression of Nectin-4 significantly promotes the proliferation of cancer cells and contributes to the intra-tumoral angiogenesis, and it can also be used as an important prognostic predictor for the patients [9]. The underlying molecular mechanism of abnormal Nectin-4 expression in cancer progression still remains to be further investigated. Of note, it has been revealed that over-expression of Nectin-4 in cancer progression can promote the intra-tumoral angiogenesis and facilitate the tumor growth [15, 23]. In addition, the PI3K/AKT signaling pathway is involved in the Nectin-4-mediated promotion of cancer cell proliferation [25–27]. Our present data also showed that increased Nectin-4 expression could significantly enhance the cell proliferation in vitro and tumor growth in vivo. However, the detailed mechanism of Nectin-4 in promoting cancer progression still deserved further investigation.

Collectively, our present findings suggested that over-expression of Nectin-4 promoted the EC progression, and such up-regulation was correlated with the poor prognosis of the patients, and Nectin-4 could serve as a useful prognostic predictor and an important therapeutic target against EC.

## Conclusions

Our present data unveiled that Nectin-4 played an important role in tumor biology and could serve as a useful prognostic predictor of human EC.

## Abbreviations

EC: esophageal cancer
IHC: immunohistochemistry
H&E: hematoxylin and eosin
FBS: fetal bovine serum
DMEM: Dulbecco’s modified Eagle’s medium
PBS: phosphate-buffered saline
shRNA: small hairpin RNA
